# Melody Processing Characterizes Functional Neuroanatomy in the Aging Brain

**DOI:** 10.3389/fnins.2018.00815

**Published:** 2018-11-19

**Authors:** Jennifer L. Agustus, Hannah L. Golden, Martina F. Callaghan, Rebecca L. Bond, Elia Benhamou, Julia C. Hailstone, Nikolaus Weiskopf, Jason D. Warren

**Affiliations:** ^1^Dementia Research Centre, Department of Neurodegenerative Disease, UCL Queen Square Institute of Neurology, University College London, London, United Kingdom; ^2^Wellcome Trust Centre for Neuroimaging, UCL Queen Square Institute of Neurology, University College London, London, United Kingdom; ^3^Department of Neurophysics, Max Planck Institute for Human Cognitive and Brain Sciences, Leipzig, Germany

**Keywords:** aging, complex sound, fMRI, music, semantic, temporal

## Abstract

The functional neuroanatomical mechanisms underpinning cognition in the normal older brain remain poorly defined, but have important implications for understanding the neurobiology of aging and the impact of neurodegenerative diseases. Auditory processing is an attractive model system for addressing these issues. Here, we used fMRI of melody processing to investigate auditory pattern processing in normal older individuals. We manipulated the temporal (rhythmic) structure and familiarity of melodies in a passive listening, ‘sparse’ fMRI protocol. A distributed cortico-subcortical network was activated by auditory stimulation compared with silence; and within this network, we identified separable signatures of anisochrony processing in bilateral posterior superior temporal lobes; melodic familiarity in bilateral anterior temporal and inferior frontal cortices; and melodic novelty in bilateral temporal and left parietal cortices. Left planum temporale emerged as a ‘hub’ region functionally partitioned for processing different melody dimensions. Activation of Heschl’s gyrus by auditory stimulation correlated with the integrity of underlying cortical tissue architecture, measured using multi-parameter mapping. Our findings delineate neural substrates for analyzing perceptual and semantic properties of melodies in normal aging. Melody (auditory pattern) processing may be a useful candidate paradigm for assessing cerebral networks in the older brain and potentially, in neurodegenerative diseases of later life.

## Introduction

The functional neuroanatomical substrates of cognitive function in the normal older brain are of considerable interest but remain poorly understood. Auditory processing is an attractive model system for studying brain mechanisms of cognition during aging, on several grounds. Sounds can be presented as auditory patterns over different time-scales and levels of analysis, ranging from early perceptual encoding to abstract, symbolic or ‘semantic’ processing in which sounds become invested with associated meaning (Goll et al., 2010). Most pertinently, understanding cerebral mechanisms of sound processing in the older brain is essential to interpret cerebrally based changes in hearing function that may presage cognitive decline and determine adjustment to complex auditory environments during normal as well as pathological aging and dementia (Hardy et al., 2016). Brain mechanisms of sound processing are likely to be sensitive to the effects of normal aging: available psychoacoustic and functional neuroimaging evidence suggests that aspects of auditory scene analysis, auditory object encoding and the processing of complex auditory patterns are altered in older listeners (Kovacevic et al., 2005; Snyder and Alain, 2007; Hailstone et al., 2009; Wong et al., 2009; Peelle et al., 2010; Rimmele et al., 2012; Cliff et al., 2013; Profant et al., 2015; Sikka et al., 2015).

While speech is the paradigm for information processing in complex auditory signals, other patterned, complex sounds are likely to make comparable demands on cognitive and neural resources in the older brain. Paramount among these is music: a complex auditory stimulus with separable perceptual and associative dimensions (Peretz et al., 1994; Peretz and Coltheart, 2003). Music has well-established structures and rules that are internalized implicitly by all normal listeners, based on prior musical exposure. Moreover, music is universal and ubiquitous and serves important social and emotional ends; the potential role of music in promoting or salvaging brain function in healthy older people as well as people with dementia has attracted much recent interest (Clark and Warren, 2015). The brain mechanisms that process music offer a window on aging cerebral function that complements the more widespread emphasis on mnestic, executive, and linguistic processing. Functional neuroimaging evidence in the young healthy brain (Platel et al., 1997, 2003; Besson and Schön, 2001; Satoh et al., 2006; Plailly et al., 2007; Groussard et al., 2010a,b) suggests that the brain mechanisms that process music are likely to be even more widely distributed than those engaged by language, instantiated in distributed brain networks spanning the temporal, frontal and parietal lobes of both cerebral hemispheres. However, there are currently few data on the functional cerebral substrates of music information processing in normal older listeners (Sikka et al., 2015). Further, establishing functional correlates of information processing in the distributed neural networks that process music would facilitate comparisons with neurodegenerative disease states in which these networks are selectively targeted (Zhou et al., 2010; Pievani et al., 2011; Warren et al., 2013a). Profiling the normal physiology of cerebral networks in later life might help predict brain functions that are resilient as well as vulnerable to neurodegenerative pathologies. Music is a case in point: it is likely that at least some aspects of memory for music are relatively spared in Alzheimer’s disease, however candidate functional neuroanatomical mechanisms have been extrapolated from the younger brain (Jacobsen et al., 2015), while other neurodegenerative diseases are characterized by distinct profiles of impairment and resilience of particular musical functions (Omar et al., 2012; Hardy et al., 2016).

In this study, we used functional MRI (fMRI) to investigate brain substrates of generic, non-verbal auditory information processing in normal older individuals, using of musical melodies. An important subsidiary aim of the study was to establish proof of principle for a paradigm that could be adapted in future to the study of older patients with neurodegenerative brain pathologies. In designing the fMRI paradigm, we manipulated orthogonally two essential attributes of melodies: temporal structure and prior familiarity. These two factors were selected to constitute a low-level, inherent perceptual property (temporal or rhythmic structure) and a higher level, associative sound property derived from previous auditory experience (melodic familiarity). The analysis of temporal structure in melodies recruits brain mechanisms that represent dynamic perceptual characteristics of auditory objects (Teki et al., 2011a): these mechanisms are likely to be critical for the subsequent identification of melodies. Processing the familiarity of melodies engages brain mechanisms of semantic memory that associate these sound objects with meaning (Groussard et al., 2010a). Previous functional imaging evidence has suggested that these musical factors are processed by separable components of a distributed, predominantly ventrally directed brain network: temporal structure is processed by posterior superior temporal, inferior parietal, and prefrontal cortices and their subcortical connections (Grahn and Brett, 2007; Bengtsson et al., 2009; Grahn and Rowe, 2009; Teki et al., 2011a,b; Marchant and Driver, 2013); while familiarity is processed by more anterior superior temporal, temporal polar and inferior frontal and opercular cortices (Besson and Schön, 2001; Platel et al., 2003; Satoh et al., 2006; Plailly et al., 2007; Groussard et al., 2010a,b; Sikka et al., 2015). The planum temporale has been characterized as a ‘computational hub’ for processing and integrating these different kinds of auditory information (Griffiths and Warren, 2002). Such evidence suggests that the manipulation of temporal structure and familiarity in melodies may be a useful approach for assessing distributed functional neuroanatomical substrates of auditory pattern processing in the older brain. Here, we employed a passive listening paradigm that did not depend on an output task or directed attention, to allow characterization of essential mechanisms of generic auditory pattern analysis as embodied in melodies. Passive listening more closely reflects the typical musical experience of musically untrained listeners in everyday life; moreover, with a view to future, clinical applications of similar paradigms (for example, in patients with dementia), it will be desirable to minimize potentially confounding effects from task difficulty or cognitive load.

A key issue in functional imaging of human auditory cortex is the relationship of functional measures to underlying neural architecture: auditory cortical areas such as the primary auditory cortex cannot be reliably delineated *in vivo* using conventional structural landmarks due to wide individual variation in cortical macroscopic and functional topographies. However, high-resolution, quantitative structural MR protocols that measure parameters sensitive to the tissue microstructural environment can be used to define cortical areas *in vivo*, in combination with fMRI (Dick et al., 2012; Lutti et al., 2014). One such parameter is magnetization transfer saturation (MT): unlike standard T1 relaxation, MT is a semi-quantitative measure of water content in brain tissue that is more direct measure of myelin integrity and allows more accurate assessment of intracortical fine structure. Here, we measured MT (adapting the protocol of Dick et al., 2012) to assess whether intracortical myelin integrity in early auditory cortex (Heschl’s gyrus) and a representative higher-order auditory cortical region (planum temporale) is linked to fMRI responses to relevant auditory parameters (sound stimulation and temporal variation, respectively).

Our hypotheses in this study were threefold. Based on previous fMRI evidence, we hypothesized, firstly, that melodies would engage a distributed cortico-subcortical network (including bilateral auditory cortices, ascending auditory pathways, insula and prefrontal cortices) in normal older participants. Secondly, and more specifically, we hypothesized that analysis of core perceptual and associative musical properties would have separable functional neuroanatomical signatures, centered on posterior superior temporal cortices for the processing of rhythmic (temporal) structure and engaging anterior temporal, and inferior frontal and parietal cortices for the processing of melodic familiarity (musical semantic memory). Thirdly, we hypothesized that functional responses in auditory cortex would positively correlate with underlying tissue myeloarchitectural integrity, indexed using MT: Dick et al., 2012).

## Materials and Methods

### Participants

Twenty healthy older individuals (56–78 years old; mean age 66.6 years; 10 females; one left-handed) with no previous history of hearing abnormalities, neurological or psychiatric illness gave written informed consent to participate in this study. None of the participants had evidence of pathological atrophy, significant vascular disease or other abnormalities on structural brain MRI performed as part of the study. All participants scored a minimum of 27/30 on the Mini-Mental State Examination (mean 29 ± 0.9 SD). Fourteen of 20 participants (seven female) had pure tone audiometry confirming normal peripheral hearing function (details in Supplementary Material on-line).

The study was approved by the local institutional research ethics committee (National Hospital for Neurology and Neurosurgery, and Institute of Neurology Joint Research Ethics Committee) and all participants gave written informed consent in line with the Code of Ethics of the World Medical Association (Declaration of Helsinki).

### Experimental Paradigm

A 2 × 2 factorial design (familiarity × temporal structure) was used to generate four conditions: (i) familiar melodies with isochronous note durations, **FI**; (ii) familiar melodies with anisochronous note durations, **FA**; (iii) unfamiliar melodies with isochronous note durations, **UI**; (iv) unfamiliar melodies with anisochronous note durations, **UA**. A further condition comprising silence trials was included as a low-level baseline. Trials were presented in a pseudo-randomized order and each condition contained 36 trials (18 trials per condition in each experimental run; 180 trials in toto). A fixed presentation order was chosen in view of potential application to a patient population as single case studies.

### Experimental Stimuli

**FIGURE 1:**
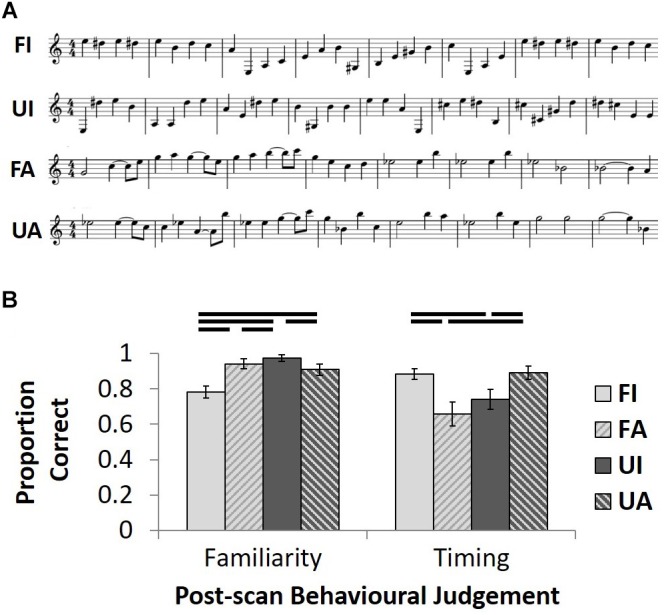
Stimulus exemplars and post-scan behavioral test results. **(A)** A 2x2 design manipulated the familiarity (**F**amiliar/**U**nfamiliar) and temporal structure (**I**sochronous**/A**nisochronous) of the musical stimuli. Unfamiliar melodies (**UI** and **UA**) were generated by randomly assigning note pitches from the familiar melodies (**FI** and **FA**) whilst maintaining the same temporal structure as familiar melodies. Examples of familiar melodies shown are taken from Beethoven’s Fur Elise (**FI**) and Prokofiev’s Peter and the Wolf (**FA**). **(B)** Group mean proportion correct ( ± 1 SEM) for post-scan familiarity and temporal judgment tasks plotted for each of the four conditions.

Familiar melodies comprised 48 excerpts from tunes widely known among older British people (see Supplementary Tables S1, S2; Figure 1A). Popular classical instrumental (non-vocal) tunes with minimal verbal associations were chosen to reduce any effects from verbal labeling. In a stimulus selection pilot study, each individual melodic excerpt selected was classified as familiar (versus unfamiliar) by at least four of five healthy older native British listeners (all >50 years of age, none of whom participated subsequently in the fMRI study; see Supplementary Tables S1, S2). Notes within 24 of the familiar melodies chosen had either fixed, isochronous durations, or almost-regular durations requiring minimal manipulation to achieve isochrony (**FI** condition*;* Supplementary Table S1). The remaining 24 familiar melodies had notes with varying, anisochronous durations (**FA** condition; Supplementary Table S2). Anisochronous and isochronous melodies were comparable in overall mean tempo and pitch variation (see Supplementary Tables S1, S2). Forty-eight unfamiliar melodies (24 isochronous, **UI**; 24 anisochronous, **UA**) were created by randomly re-distributing the pitch values within each single excerpt from the familiar melody trials, such that each note used in the familiar trials was reused once in composing the unfamiliar melodies (see Figure 1A). This resulted in each familiar melodic excerpt having an unfamiliar counterpart that retained the same temporal structure (conforming to musical bar timing). As a measure of the overall ‘tunefulness’ of the familiar and unfamiliar melody conditions, we calculated the information content (entropy) standard error of the pitch sequences in each condition using IDyOM software for predictive statistical modeling of music structure (Pearce, 2005; Pearce and Wiggins, 2012): this did not differ significantly between conditions (*F*-statistic 0.427, *p* = 0.515). However, our condition manipulation produced unavoidable alterations in harmonic structure and key. For each of the four conditions, 12 of the stimuli were presented once and the other 12 stimuli were presented twice to create a total of 36 trials across per condition across the two experimental runs. Presentation orders were pseudo-randomized to ensure two identical trials did not follow directly each other.

Stimuli were generated as digital wavefiles using MATLAB v7.0 (MathWorks, Inc., Natick, MA, United States) with sampling rate 44.1 kHz. Individual notes were harmonic complexes (f1, 3, 5, 7, 9) based on the target pitch with a 6 ms cosine ramp at onset and offset. Notes were concatenated into sequences each lasting 8 s and containing on average 29.5 notes (range 16–48 notes; see Supplementary Tables S1, S2) for each of the four conditions. Successive notes were separated by a 6 ms gap. The average intensity (rms value) of each trial was fixed across conditions. Examples of the stimuli are provided in Supplementary Material.

### Brain Image Acquisition Protocol

Auditory stimuli were presented under Cogent v1.28 software (Vision Lab, University College London, United Kingdom) running in MATLAB and delivered binaurally using electro-dynamic headphones (MR Confon GmbH, Magdeburg) at a fixed comfortable listening level (approximately 70 dB sound pressure level). An exemplar melodic excerpt was presented to each participant once positioned inside the MR scanner bore, to ensure the stimuli were clearly audible in both ears. Participants were instructed to listen to the sound stimuli and to keep their eyes open; no output task was administered and no participant responses were required during the scanning sessions.

Brain images were acquired on a 3T TIM Trio whole-body MRI scanner (Siemens Healthcare, Erlangen, Germany) using a 12-channel RF receive head and body transmit coil. For the two functional runs, 92 single-shot gradient-echo EPI (echo-planar image) volumes were acquired with 48 oblique transverse slices with slice thickness 2 mm, inter-slice gap 1 and 3 mm in-plane resolution (slice time = 70 ms; echo time TE = 30 ms; echo spacing = 0.5 ms; matrix size = 64 × 64 pixels; FoV = 192 mm × 192 mm, phase encoding [PE] direction anterior–posterior). A slice tilt of -30° (T > C), z-shim gradient moment of +0.6 mT/m^∗^ms and positive PE gradient polarity were used to minimize susceptibility-related loss of signal and blood-oxygen-level-dependent (BOLD) functional sensitivity in the temporal lobes, following optimization procedures described previously (Weiskopf et al., 2006). A sparse-sampling EPI acquisition paradigm with repetition time (TR) of 11.36 s was used to reduce any interaction between scanner acoustic noise and auditory stimulus presentations (Agustus et al., 2015). Each functional run was 17.3 min in duration. The initial two brain volumes were discarded from analysis to allow for equilibrium of the longitudinal magnetization (T1 equilibrium). A 2 min B0 field-map was acquired using two gradient echo sequences (TE1 = 10 ms, TE2 = 12.46 ms, 3 mm × 3 mm × 2 mm resolution, 1 mm gap; matrix size = 64 × 64 pixels; FoV = 192 mm × 192 mm) to allow for post-processing correction of geometric distortion corrections of the EPI data due to B0 field inhomogeneity.

A multi-parameter mapping (MPM) protocol lasting approximately 10 min was used to acquire 1 mm isotropic volumetric structural brain MR images (Weiskopf et al., 2013). This comprised three spoiled 3D multi-echo FLASH scans with predominantly proton density (PDw: TR = 23.7 ms; flip angle = 6°), T1 (T1w: 18.7 ms; 20°) and magnetization transfer weightings (MTw: 23.7 ms; 6°; Weiskopf et al., 2013). Six alternating gradient echoes were acquired at equidistant echo times for the T1w and MTw sequences, and eight for the PDw sequence (FoV = 256 mm × 240 mm × 176 mm; GRAPPA factor 2 in PE direction and 6/8 Partial Fourier factor in partition direction). In addition, a 3D EPI acquisition of spin and stimulated echoes (SE/STE) with different refocusing flip angles (TE_SE_ = 37.06 ms; TE_STE_ = 68.26 ms; TM = 31.20 ms; TR = 500 ms; matrix = 64 ms × 48 ms × 48 ms; FoV = 256 mm × 192 mm × 192 mm) was acquired to estimate the local RF transmit field (Helms et al., 2008; Lutti et al., 2010).

All participants were in the MR scanner for a maximum of 1 h, with pauses of several minutes between scanning protocols. The scanning protocols were presented in the same fixed order for all participants with the two functional runs followed by the B0 fieldmap and then the MPM anatomical scan. There was a break of at least 15 min before conducting post-scan behavioral tests (described below). All parts of the study were conducted on the same day.

### Post-scan Behavioral Assessments

After scanning participants were asked to complete a questionnaire detailing their prior and current musical experience, including years spent learning or playing any instrument, hours per week spent listening to music and preferred genre of music (Hailstone et al., 2009). Half (10) of the participants reported never having played a musical instrument or undertaken any form of musical training; only four participants reported that they still played an instrument on a regular basis, and none was a professional musician. Most (six of the seven) participants who reported playing an instrument for at least 1 year had played the piano. Participants reported listening to 5.75 h of music per week on average, although across the participant group there was a broad range of time spent listening to music (0–25 h) and preferred genre of music (e.g., jazz, pop, rock, easy listening, classical).

Participants were also assessed for their ability to discriminate the experimental conditions presented during scanning using post-scanner behavioral tests. A small subset of 24 experimental auditory stimuli (six trials from each of the four sound conditions) were presented in fixed, pseudo-randomized order in two short testing sessions of 6 min duration each in a succinct procedure that could readily be applied to a patient population in view of potential future clinical application of this study. In the first test, familiarity judgment, participants were asked to decide whether each tune was familiar or unfamiliar; in the second test, temporal judgment, they were asked to decide whether the notes composing each tune were of fixed (isochronous) or varying (anisochronous) duration. Participant responses were recorded for off-line analysis. For each test, the proportion of correct responses was calculated for the four experimental conditions for each participant and entered into a 2 × 2 within-participant ANOVA (familiarity × temporal judgment) for statistical analysis using SPSS v16.0 (SPSS, Inc., Chicago, IL, United States). *Post hoc* paired *t*-tests were used to interrogate any significant interaction effects (*p* < 0.05).

### Analysis of Brain Imaging Data

Brain imaging data were analyzed using statistical parametric mapping software (SPM8^1^). In light of the age range of the participant group here, a group-specific structural template brain image was generated. The MPM maps were estimated from the multi-echo FLASH scans using the VBQ toolbox (Draganski et al., 2011) in SPM8 and the resulting MT map for each participant was used in the further processing (Weiskopf et al., 2011; Lutti et al., 2013). MT maps were coregistered to the functional images, segmented and entered into the DARTEL toolbox to create a group template image that was aligned to Montreal Neurological Institute (MNI) standard space (Ashburner, 2007). fMRI scans for each participant were realigned using the first image as a reference, and unwarped incorporating field-map distortion information (Hutton et al., 2002). DARTEL processing was used to spatially normalize individual fMRI scans to the group mean template image in MNI space. Normalized fMRI images were smoothed using a 6 mm full-width at half-maximum Gaussian smoothing kernel.

Pre-processed functional images were entered into a first-level design matrix incorporating the four experimental conditions (**FI**, **FA**, **UI**, **UA**) modeled as separate regressors with boxcars of one TR duration convolved with the canonical hemodynamic response function, and six head movement regressors derived from the realignment process. First-level contrast images were generated for the main effects of auditory stimulation (**[FI + FA + UI + UA] > silence**), temporal structure (**[FI + UI] vs. [FA + UA]**), familiarity (**[FI + FA] vs. [UI + UA]**), and the interaction between the two experimental factors (**[FI > FA] vs. [UI > UA]**). Contrast images for each participant were entered into a second-level random-effects analysis using *t*-tests with covariates for age, years spent playing an instrument and average hours per week spent listening to music (indices of musical experience derived from the behavioral questionnaire). For the familiarity contrast, additional covariates for overall proportion correct and effect of familiarity condition on post-scan performance derived from the post-scan familiarity judgment task were included in the second-level analysis. For the temporal contrast, additional covariates for overall proportion correct and effect of temporal condition on post-scan performance derived from the post-scan timing judgment task were included in the second-level analysis.

The correlation coefficient between BOLD response (t-score) for the main effects of auditory stimulation (**[FI + FA + UI + UA] > silence**) and MT value was calculated for each participant across all voxels within the regions of interest (ROI) in early auditory areas (Heschl’s gyrus, HG) bilaterally. This correlation analysis was repeated for voxels within ROI in higher auditory areas [planum temporale (PT)] for the main effect of temporal structure (**[FI + UI] > [FA + UA]**). ROIs were derived from the Oxford-Harvard brain maps (Desikan et al., 2006) in fslview (Jenkinson et al., 2012) and used to extract gray matter maps for each participant. A Fisher transformation was applied to the correlation coefficient estimated for each participant, in order to normalize the correlation coefficient distribution and homogenize its variance and condition appropriately for further statistical tests. A one-tailed *t*-test was used to test our hypothesis that there is a positive correlation between MT value and BOLD response across participants (*p* < 0.01).

## Results

### Behavioral and Background Data

In the familiarity judgment task, there was a main effect of melodic familiarity (*F*_1,19_= 25.0; *p* < 0.001) and an interaction between this factor and temporal condition (*F*_1,19_= 24.2; *p* < 0.001) on performance (Figure 1B); *post hoc* paired *t*-tests on the familiarity task (employed since the data on this task were found not to differ significantly from normality after arcsine transformation to account for ceiling effects) revealed that participants correctly classified isochronous, unfamiliar tunes most accurately (UI > UA: *t*_19_ = 2.6, *p* = 0.017; UI > FA: *t*_19_ = 2.4, *p* = 0.025; UI > FI: *t*_19_ = 6.5, *p* < 0.001) and isochronous familiar tunes least accurately (UA > FI: *t*_19_ = 4.0, *p* = 0.001; FA > FI: *t*_19_ = 4.4, *p* < 0.001), while accuracy of familiarity judgments did not differ for the two anisochronous conditions (FA > UA: *t*_19_ = 0.6, *n.s.*). For performance on the temporal judgment task, there was an interaction between the two factors of melodic familiarity and temporal condition (*F*_1,19_ = 14.1; *p* = 0.001; Figure 1B); *post hoc* paired *t*-tests revealed a cross-over interaction, whereby familiarity enhanced and unfamiliarity reduced correct classification of isochronous melodies when compared to anisochronous melodies (FI > UI: *t*_19_ = 2.2, *p* = 0.039; FA < UA: *t*_19_ = 3.7, *p* = 0.001); familiar anisochronous melodies were more difficult to classify correctly than familiar isochronous melodies (FI > FA: *t*_19_ = 2.7, *p* = 0.014).

### fMRI Data

**FIGURE 2:**
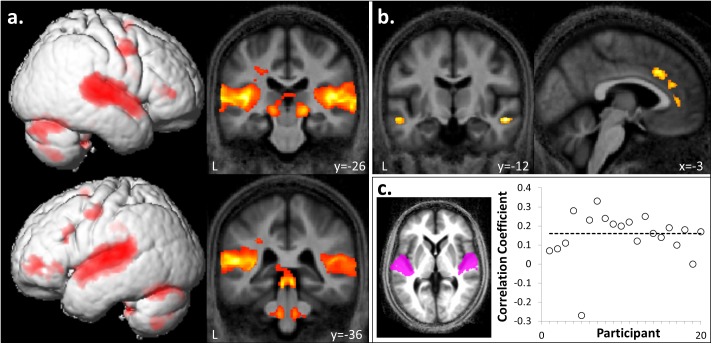
Whole brain responses to auditory stimulation and correlation with intracortical myelination. **(a)** A bilateral auditory brain network, including superior colliculi, lateral geniculate nuclei, superior temporal, and inferior frontal gyri was activated during passive listening to sound compared to silence (**[FI + FA + UI + UA] > silence**). **(b)** In contrast, anterior cingulate, bilateral anterior superior temporal sulci and cuneus showed relatively more activation during silence (**[FI + FA + UI + UA] < silence**). Significant clusters (cluster p_FWE_< 0.05, voxel p_unc_< 0.001) are displayed on the group mean MT map normalized to a group template brain image in Montreal Neurological Institute (MNI) standard space. **(c)** Plot of correlation coefficients between magnetization transfer values (a metric of intracortical myelin integrity) and BOLD response (t-score) in Heschl’s gyrus (HG) for the auditory stimulation contrast collapsed across hemispheres, for individual participants (*n* = 20). The mean corrected correlation coefficient is indicated by the horizontal line. Inset, HG regions of interest displayed on the group mean MT map normalized to a group template brain image in MNI space.

**FIGURE 3:**
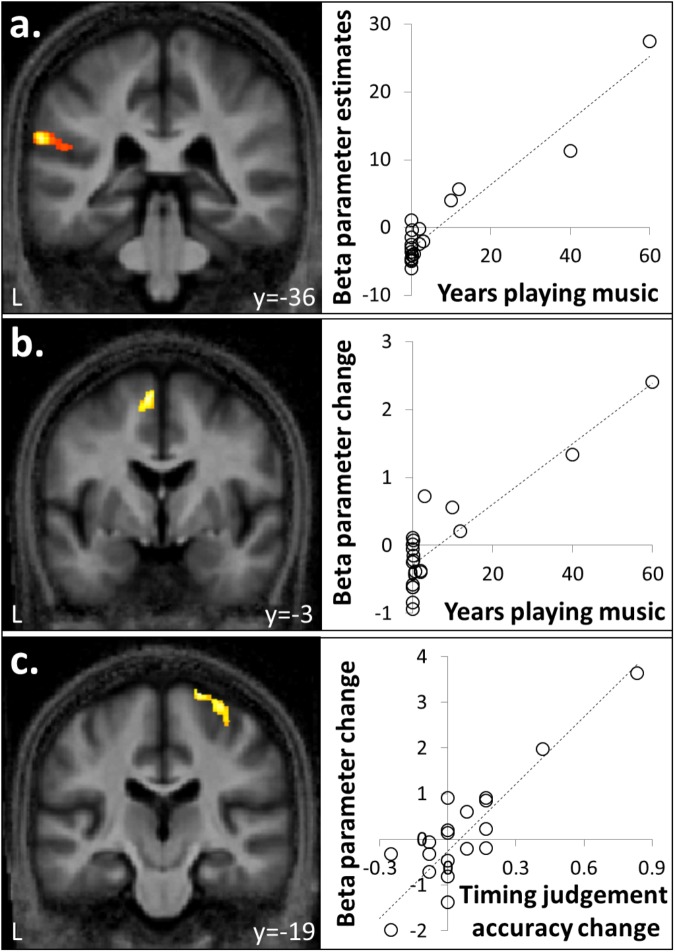
Correlation between brain responses to auditory stimuli and musical experience or timing judgment. **(a)** Activity in left supramarginal gyrus associated with auditory stimulation was positively correlated with number of years spent playing an instrument. **(b)** Activity in supplementary motor area (SMA) for familiar compared to unfamiliar melodies was a positively correlated with years spent playing a musical instrument. **(c)** Activity in right premotor cortex for isochronous compared to anisochronous melodies (**[FI + UI] > [FA + UA]**) was positively correlated with the effect size of isochrony on post-scan temporal judgment performance. One data-point plotted per participant for peak cluster beta parameter estimate (*n* = 20).

**FIGURE 4:**
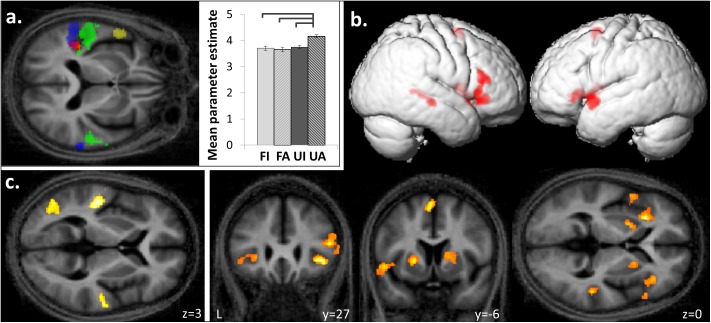
Functional segregation within planum temporale and superior temporal gyrus based on temporal structure and familiarity of melodies. **(a)** Posterior superior temporal gyrus and planum temporale bilaterally were activated by anisochronous compared to isochronous melodies (**[FI + UI] < [FA + UA]**, blue) and by unfamiliar compared to familiar melodies (**[FI + FA] < [UI + UA]**, green). An interaction between the familiarity and temporal structure factors was observed in left medial planum temporale (**[FI < FA] < [UI < UA]**; red) and the group mean cluster beta parameter estimates plots (±1 SD for timing) show a greater response to unfamiliar-anisochronous melodies (**UA**) than the other three conditions (significant *p* < 0.05). Left anterior temporal lobe showed more activation to familiar than unfamiliar melodies (**[FI + FA] > [UI + UA]**, yellow). **(b)** Familiar melodies also preferentially activated right superior temporal sulcus, and bilateral inferior frontal gyri, insula, putamen, and SMA (**[FI + FA] > [UI + UA]**). **(c)** Unfamiliar compared to familiar melodies activated the left lateral occipito-temporal cortex in addition to the bilateral temporal gyri (**[FI + FA] < [UI + UA]**). Significant clusters (cluster p_FWE_ < 0.05, voxel p_unc_ < 0.001) are displayed on the group mean MT map normalized to a group template brain image in MNI space. In figure only, the transverse MNI section is pitched –0.5 radians to better visualize activation along the superior temporal gyri.

Statistical parametric data derived from the fMRI analysis are presented in Figures 2–4 and Tables 1, 2. All significant voxel clusters are reported at cluster threshold p_FWE_ < 0.05 corrected for multiple comparisons over the whole brain volume (voxel-wise threshold p_unc_< 0.001 uncorrected) and in MNI space.

**Table 1:** Brain regions activated during passive listening to auditory stimuli compared to silence.

	Left hemisphere						Right hemisphere					
		
	*Cluster*		*Peak voxel*				*Cluster*		*Peak voxel*			
				
	*p_FWE_*	*Voxels*	*t_20_*	*x*	*y*	*x*	*p_FWE_*	*Voxels*	*t_20_*	*x*	*y*	*z*
**(A) *All auditory stimuli > silence***												
Inferior frontal gyrus	<0.001	861	6.84	-51	47	-2	0.026	225	5.73	43	42	3
Dorsolateral prefrontal cortex	0.022	233	5.99	-51	20	30						
Supplementary motor area							0.002	352	6.82	11	0	63
Superior temporal gyrus	<0.001	8817	14.85	-45	-19	4	<-0.001	8379	16.91	59	-13	0
Premotor cortex	0.002	348	7.97	-50	-4	46	<0.001	679	9.16	57	2	45
Inferior colliculus	<0.001^a^	2256	11.22	-5	-36	-12	^a^		11.02	3	-39	-9
Medial geniculate nucleus	^a^		7.35	-14	-25	-5	^a^		8.65	17	-25	-6
Olivary nucleus	0.009	280	7.02	-8	-37	-42	0.047	197	7.17	8	-36	-42
Thalamus	0.001	381	6.11	14	-10	12						
Cerebellum (VI)	<0.001	2029	9.60	-27	-63	-23	<0.001	2670	12.39	27	-64	-23
Cerebellum (VIII)	<0.001	613	9.52	18	-69	-53	<0.001	448	8.27	-21	-66	-54
**(B) *Silence > auditory stimuli***												
Anterior cingulate cortex	0.013	258	6.25	-3	20	34						
Medial prefrontal cortex	<0.001	462	6.67	-3	41	4						
Superior temporal sulcus							0.004	324	8.22	53	-12	-15
Cuneus	0.018	243	5.81	-20	-67	27	0.007	289	6.04	24	-63	22


*Significant clusters (p_*FWE*_< 0.05) reported. ^*a*^Local peaks for nuclei within a single larger cluster.*

**Table 2:** Brain regions activated during the different auditory conditions used in this experiment.

		*Cluster*		*Peak voxel*			
			
		*p_FWE_*	*Voxels*	*t_20_*	*x*	*y*	*x*
**(A) *Anisochronous > isochronous melodies: [FI + UI] < [FA + UA]***							
L	Superior temporal gyrus	<0.001	904	10.11	-60	-39	6
R	Superior temporal gyrus	<0.001	660	8.51	68	-30	13
**(B) *Familiar > unfamiliar melodies: [FI + FA] > [UI + UA]***							
R	Inferior frontal gyrus	<0.001	1025	9.76	44	29	-6
L	Insula/inferior frontal gyrus	0.002	367	10.02	-29	23	0
L	Temporal pole	<0.001	454	6.31	-51	6	-5
*R*	*Temporal pole*	*^a^0.208*	*133*	*8.14*	*56*	*11*	-*17*
R	Superior temporal sulcus	0.002	366	8.98	47	-22	-9
-	Supplementary motor area	0.008	293	7.53	-2	5	63
L	Putamen	0.011	274	6.98	-21	6	3
R	Putamen	0.001	408	5.46	17	8	6
**(C) *Unfamiliar > familiar melodies: [FI + FA] < [UI + UA]***							
L	Superior temporal gyrus	<0.001	1293	7.19	-48	-22	3
R	Superior temporal gyrus	<0.001	590	6.97	66	-15	10
L	Precuneus	0.031	222	5.48	-8	-55	31
L	Lateral occipito-temporal cortex (V5)	0.001	419	6.04	-47	-69	1
**(D) *Familiarity × temporal interaction: [FI < FA] < [UI < UA]***							
L	Superior temporal gyrus	0.035	205	6.12	-41	-34	15


*Significant clusters (p_*FWE*_< 0.05) reported for each comparison. *^*a*^*Subthreshold clusters of interest.*

#### fMRI Data: Auditory Stimulation

Auditory stimuli compared to the silence baseline (**[FI + FA + UI + UA] > silence**) engaged a distributed brain network including subcortical auditory structures (olivary bodies, inferior colliculi, medial geniculate nuclei) and cerebellum together with bi-hemispheric cortical regions including medial and lateral Heschl’s gyrus (HG), planum temporale (PT), posterior superior temporal gyrus (STG) and temporo-parietal junction and extending dorsally to premotor cortices, supplementary motor area (SMA) and inferior frontal gyrus (Figure 2a and Table 1A). BOLD responses in supramarginal gyrus associated with auditory stimulation were significantly positively correlated with previous musical performance (years spent playing an instrument) (*cluster: p_FWE_* = 0.023, *n* = 232*; peak voxel: t* = 13.25, *x* = -62, *y* = -34, *z* = 24; Figure 3a); no brain regions showed activity correlated with participant age or current musical exposure (time spent listening to music each week). This correlation was driven by the two most musically experienced participants, and was no longer significant after these were excluded. The reverse contrast (**[FI + FA + UI + UA] < silence**) was associated with significant differential activation of anterior STS, anterior cingulate gyrus, and cuneus (Figure 2b and Table 1B).

#### fMRI Data: Temporal Structure of Melodies

Anisochronous melodies produced significantly more activation than isochronous melodies (**[FI + UI] < [FA + UA]**) in posterior STG and PT bilaterally (Figure 4a and Table 2A). The reverse contrast (**[FI + UI] > [FA + UA]**) produced no significant activations. However, activity in right premotor cortex for this contrast was significantly positively correlated with the effect size of isochrony on post-scan temporal judgment performance (*cluster: p_FWE_* = 0.045, *n* = 175; *peak voxel: t* = 6.41, *x* = 26, *y* = -19, *z* = 72; Figure 3c).

#### fMRI Data: Familiarity of Melodies

Familiar melodies produced significantly more activation than unfamiliar melodies (**[FI + FA] > [UI + UA]**) in left temporal pole, right superior temporal sulcus (STS), and bilaterally in insula and putamen and extending into the inferior frontal gyri and SMA (Figures 4a,b and Table 2B); the right temporal pole showed less robust activation that did not reach statistical significance after multiple comparisons correction (p_FWE_ = 0.208). Activity in SMA associated with processing familiar melodies was significantly positively correlated with previous musical experience (years spent playing an instrument) (*cluster: p_FWE_* = 0.007, *n* = 295; *peak voxel: t* = 6.52, *x* = -6, *y* = -7, *z* = 70; Figure 3b); once more this correlation was no longer significant after the two most experience musicians were excluded. No brain regions showed activity correlated with performance in the post-scan familiarity judgment task. The reverse contrast (**[FI + FA]** **< [UI + UA]**) produced significant activation in left lateral HG, posterior STG and PT bilaterally, precuneus and lateral occipito-temporal cortex (in the region of human V5 complex; Watson et al., 1993) (Figures 4a,c and Table 2C). Activity in PT was more antero-medial than the activation observed for the temporal structure contrast (Figure 4a and Table 2).

#### fMRI Data: Interaction Between Familiarity and Temporal Structure

There was a significant interaction between the two experimental factors (familiarity × temporal structure) in left medial PT (Figure 4a and Table 2D): this interaction effect was in the direction of a greater impact of anisochrony on processing of unfamiliar than familiar melodies (**[FI < FA] < [UI > UA]**). *Post hoc* analysis of mean cluster beta values confirmed that this effect was driven by a significantly greater response to unfamiliar anisochronous melodies than melodies in other conditions (all comparisons *p* < 0.05; Figure 4a).

### Correlation Between BOLD Response to Sounds and Myelination in Auditory Cortex

Magnetization transfer saturation values in HG across right and left hemispheres were significantly positively correlated with BOLD responses in the auditory stimulation contrast (**[FI + FA + UI + UA] > silence**) with corrected mean correlation coefficient across participants 0.16 (*p* < 0.001) (Figure 2c); a significant positive correlation was also present for each hemisphere separately (left HG, coefficient 0.13; right HG, coefficient 0.19). There was no significant correlation at the specified threshold (*p* > 0.01) between MT values and BOLD responses in non-primary auditory cortex in PT for the temporal structure contrast (**[FI + UI] < [FA + UA]**).

## Discussion

Here, we have demonstrated functional neuroanatomical signatures of auditory pattern processing in melodies in a normal older cohort. The findings provide new data on brain substrates of auditory processing in healthy older people and support and extend emerging models of auditory cortical organization derived from the study of younger cohorts. Auditory stimulation (compared with silence) produced activation in a distributed, bi-hemispheric network centered on primary auditory cortex, delineating the subcortical and cortical auditory pathways. Separable signatures were identified for processing of melodic temporal structure, anisochronous melodies predominantly engaging posterior STG and PT; and melodic familiarity, more familiar melodies predominantly engaging anterior temporal and inferior frontal cortices and their subcortical connections, and unfamiliar melodies predominantly engaging more posterior superior temporal and parietal cortices. Within PT, there was a partitioning of activation, a more posterior subregion showing relatively greater activation for processing anisochrony, and a more anterior subregion showing relatively greater activation for processing novelty, with an interaction between these factors in medial PT: considered together, these findings are consistent with an organizational scheme in which PT acts as a ‘hub’ region that links functional networks engaged in processing particular properties of auditory patterns. Our findings demonstrate that auditory pattern processing (as instantiated in melodies) is a candidate paradigm for delineating distributed cerebral networks in the normal older brain. We now consider these brain network signatures in more detail.

### Processing Temporal Structure in Music

The sensitivity to temporal structure in melodies demonstrated here in posterior STG and PT is consistent with previous work in young healthy individuals showing modulation of responses to auditory and visual stimuli according to the predictability of temporal structure in posterior association auditory cortices (Bischoff-Grethe et al., 2000; Costa-Faidella et al., 2011; Teki et al., 2011b; Marchant and Driver, 2013). However, available evidence does not fully resolve the precise mechanism of this sensitivity: in this study, posterior superior temporal cortex showed enhanced responses to anisochronous compared with isochronous stimuli, whereas Teki et al. (2011b) found enhanced responses to regular (beat-based) stimuli while Costa-Faidella et al. (2011) found that temporal unpredictability maintained sensitivity for auditory deviant detection in similar cortical regions.

We did not find activation of the cortico-subcortical network previously implicated in processing different aspects of temporal structure in task-dependent fMRI studies of younger adults (Grahn and Brett, 2007; Bengtsson et al., 2009; Teki et al., 2011b; Marchant and Driver, 2013). While this might indicate an effect from aging *per se*, any such interpretation must be cautious, given the differences of design between the present and previous studies. Our use of melodies with an implicit beat structure in both the isochronous and anisochronous conditions may have attenuated any differential processing by subcortical timing processors, and this may have been further influenced by the lack here of an explicit in-scanner timing task.

### Processing Familiarity of Melodies

In line with work in younger adults, and a study by Sikka et al. (2015) that directly compared familiarity responses between older and young participants, we have shown that processing of familiar (compared with unfamiliar) melodies in the normal older brain engages a distinct distributed brain network. This network includes areas previously implicated in semantic memory for music, notably anterior STG and inferior frontal cortices (Platel et al., 2003; Satoh et al., 2003, 2006; Plailly et al., 2007; Groussard et al., 2009, 2010a,b; Peretz et al., 2009; Pereira et al., 2011; Saito et al., 2012; Sikka et al., 2015). In addition to engaging temporal polar cortex, processing of musical familiarity in the present cohort engaged a more posterior region in right anterior STS in close proximity to previously identified STS correlates of musical familiarity processing (Plailly et al., 2007; Peretz et al., 2009; Saito et al., 2012). Familiar melodies here engaged additional areas including SMA and putamen (Peretz et al., 2009; Pereira et al., 2011; Saito et al., 2012), associated previously with beat-based temporal processing (Grahn and Brett, 2009; Teki et al., 2011b) but interestingly, also implicated in musical semantic memory (Jacobsen et al., 2015). These brain regions might have been engaged in anticipatory processing of an expected or implicit beat structure in the familiar (but not unfamiliar) melodies here. This interpretation would be consistent with the results of the post-scan timing judgment task: participants were more likely to judge familiar melodies (those with predictable pitch structure) as isochronous (having a more predictable temporal structure) and unfamiliar melodies as anisochronous, suggesting an interaction of cognitive predictability between temporal and familiarity dimensions in line with previous work (Boltz, 1993). An alternative, related possibility is that these areas were engaged in anticipatory auditory imagery of familiar tunes: a similar brain network has been demonstrated in healthy young individuals during anticipatory imagery of learned melodies (Leaver et al., 2009; Herholz et al., 2015). Although there was no requirement for active melody ‘learning’ in the present experiment, the familiar melodies were presented in a somewhat non-naturalistic format (i.e., reduced to a mono-vocalic sequence of synthetic timbres) in line with the overall constraints on the experimental design, and this non-canonical presentation may have activated brain regions engaged in active modeling of auditory sequences.

The increased responses to unfamiliar melodies in more posterior cortical areas shown by the present cohort has been a less consistent finding in previous studies, however, similar anatomical associations have been reported for the processing of unfamiliar over familiar songs (Pereira et al., 2011) and for analysis of musical structure over familiarity of melodies (Satoh et al., 2006). Engagement of precuneus and lateral occipito-temporal cortex in the vicinity of V5 complex by unfamiliar melodies may indicate obligatory cross-modal (visual) imagery during the analysis of musical structure (Goebel et al., 1998; Kourtzi and Kanwisher, 2000), in line with previously demonstrated correlates of melodic pitch analysis (Platel et al., 1997; Satoh et al., 2006). An important caveat on the present familiarity contrast concerns the harmonic structure of the unfamiliar melodies, which were random pitch sequences: accordingly, the familiar and unfamiliar melodies here differed according to whether they conformed to musical ‘rules’ of Western harmony, which will have been implicitly learned by (and generically familiar to) these older listeners, as well as any more specific prior exposure to particular melodies. This may in part account for the lack of correlation between functional neuroanatomical measures and melodic familiarity judgments in the post-scan behavioral task. It is a complex issue, since learned expectations about harmony and key relations might also be regarded as a component of musical semantic memory, albeit a superordinate level of musical knowledge contrasting with familiarity for specific melodies.

### Planum Temporale: Integrating Different Dimensions of Musical Processing

Engagement of PT for processing both temporal structure and familiarity of melodies here corroborates and extends previous work suggesting that PT acts as a ‘computational hub’ for the analysis of auditory patterns (Griffiths and Warren, 2002). The activation of PT by both novel pitch sequences and anisochronous sequences supports a more generic sensitivity of PT to unpredictability (or novelty) in auditory patterns (Overath et al., 2007): this sensitivity might indicate the operation of a generic, iterative neural algorithm whereby PT ‘searches’ incoming auditory information for patterns that match stored template patterns (Griffiths and Warren, 2002; Warren and Griffiths, 2003; Warren et al., 2005). This ‘search’ would be completed rapidly in the case of temporally regular or familiar melodies (i.e., a match to a prior template is achieved) whereas novel sequences would entail increased processing (i.e., iterations of the search algorithm, failing to achieve a template match; see Figure 4a). Such a mode of operation of PT would fit with a Bayesian hierarchical framework that has been proposed as a general principle of cortical computation (Friston, 2005): according to this framework, computations in PT (and elsewhere) minimize discrepancies between incoming sensory traffic and top–down predictions from higher cortical areas, and are used in turn to update those predictions. Here we have further shown a functional anatomical partitioning of PT subregions processing temporal structure (more posterior) and unfamiliarity or novelty (more antero-medial). This functional segregation of PT may map onto the well-documented heterogeneity of this large anatomical region and in particular, a previously proposed scheme according to which more posterior PT subregions process auditory properties that could be used to index auditory objects in space or time, while more anterior PT subregions process properties relevant to auditory object identity (Griffiths and Warren, 2002; Warren et al., 2005).

The present finding of a medial PT subregion showing a BOLD interaction between melodic familiarity and temporal processing provides a candidate neural substrate for integration of these musical dimensions and for routing this integrated information to the dorsal auditory cortical pathway for subsequent programming of a behavioral response (Warren et al., 2005). If the neural mechanisms that process familiarity and temporal information interact, this might drive the performance interaction observed in our behavioral data (Figure 1B). For example, recognizing a familiar melody as familiar would be relatively more difficult in the isochronous condition, because most tunes are not in fact strictly regular, so achieving a ‘match’ to the putative stored melody template might be less efficient in this condition. Conversely, assessing temporal regularity would be more difficult when melodic and temporal structures have opposing predictability (determining anisochrony with familiar melodies – determining isochrony with unfamiliar melodies) since the putative template matching algorithms for the two dimensions would ‘compete’ in this situation. It is noteworthy that a very similar medial PT subregion has been specifically implicated in mediating speech repetition (Wise et al., 2001), which demands a precise integration of temporal and auditory object (phonemic) identity from the incoming speech signal.

### Correlation Between Musical Experience and Brain Activations

We did identify certain neuroanatomical correlates of musical experience or exposure, though in this group of non-expert musicians these were relatively limited. There was a positive correlation between the effect of any auditory stimulation and prior experience of musical performance (years spent playing an instrument) in supramarginal gyrus, in accord with previous evidence that activity in this region during musical tasks is generally enhanced by musical training (Gaab and Schlaug, 2003). A positive correlation between the effect of isochrony processing and prior musical performance was identified in right premotor cortex and we tentatively speculate this may relate to implicit time keeping. Finally, a positive correlation between the effect of melodic familiarity and prior musical performance was identified in SMA: this is in line with a previously proposed role for SMA in music processing, and in particular, musical motor imagery or internal rehearsal of familiar tunes (Halpern and Zatorre, 1999; Groussard et al., 2009; Peretz et al., 2009; Pereira et al., 2011). Caution is needed in interpreting these findings: for example, the neuroanatomical correlation of familiarity processing with prior musical performance was driven by the most experienced participants in this cohort, suggesting that extensive musical expertise may induce a reorganization of cerebral processing mechanisms that does not necessarily hold for less experienced listeners. Taking this caveat into account, we interpret the relatively sparse correlates of past musical exposure in this musically non-expert cohort as evidence that musical stimuli can be used to probe generic mechanisms of sensory pattern analysis in older individuals that are not heavily dependent on specific skills or experience.

### A Prospect for the Study of Neurodegenerative Diseases

One important role for studies of this kind is to delineate signatures of brain network function in the normal older brain that could in future provide a reference for interpreting pathological alterations of network function produced by neurodegenerative diseases. It is increasingly recognized that the major neurodegenerative diseases of later life including Alzheimer’s disease and frontotemporal lobar degeneration blight specific, distributed cortico-subcortical networks spanning the frontal, temporal, and parietal lobes (Zhou et al., 2010; Fletcher and Warren, 2011; Pievani et al., 2011; Rohrer et al., 2011; Warren et al., 2013a). Furthermore, these diseases have auditory phenotypes of altered complex sound processing attributable to involvement of these networks (Hardy et al., 2016) and structural and functional MRI signatures of disease-associated auditory dysfunction in major dementias are now being delineated (Hardy et al., 2017a,b). The fMRI paradigm developed here captures activity in key components of the brain networks previously implicated in the pathogenesis of neurodegeneration and accesses generic computations of musical stimuli that are potentially susceptible to a broad range of neurodegenerative diseases (e.g., Goll et al., 2011, 2012a,b; Hsieh et al., 2011). We are therefore optimistic that this relatively concise, simple and well-tolerated paradigm could be administered to cognitively impaired participants to assess functional brain network changes in the common neurodegenerative dementias, both as a means to predict deficits and to uncover residual capacities, in musical and other complex auditory processes (Jacobsen et al., 2015; Hardy et al., 2016). fMRI can potentially detect reversible neurodegenerative effects that predate the onset of brain atrophy; moreover, in contrast to structural MRI, fMRI can identify brain regions that show heightened activity either as a compensatory response or due to loss of processing efficiency (Goll et al., 2012b; Golden et al., 2015). With respect to disease signatures, Alzheimer’s disease might be anticipated to alter auditory processing chiefly in the more posteriorly directed areas implicated in the temporal and novelty contrasts here, since these areas overlap with the temporo-parietal so-called ‘default mode’ network primarily targeted by Alzheimer pathology (Warren et al., 2012; Hardy et al., 2016). Conversely, altered processing of melodic familiarity in the more anterior cortical regions identified here might be anticipated to expose a core computational impairment in the frontotemporal lobar degenerations, as these diseases preferentially target anteriorly directed semantic and salience networks (Warren et al., 2013b; Hardy et al., 2017a,b). It will be important that fMRI studies of auditory processing in cognitively normal as well as cognitively impaired individuals calibrate for peripheral hearing function; moreover, fMRI can capture the central effects of peripheral hearing loss directly, and thereby potentially address a key unresolved issue in older health (Hardy et al., 2016; Warren and Bamiou, 2018).

Our findings further suggest that imaging of tissue microstructure (intracortical myelin) may be a useful adjunct to the interpretation of altered cortical function in future clinical applications. However, microstructural MRI techniques have yet to be widely translated to clinical settings and indeed, myeloarchitectural parameters of normal neural aging have not been defined. The distinct myeloarchitecture of primary auditory cortex within HG may make it a particularly suitable target for such studies (Dick et al., 2012; Sereno et al., 2013). The present data suggest that primary auditory cortex may exhibit a closer correspondence between measures of microstructural (MT) and functional (BOLD) integrity than auditory association cortex: this may reflect the distinctive inter-areal connectivity as well as micro-architectural profiles of these cortices (Guéguin et al., 2007), which would tend to modulate any intrinsic synaptic alterations. However, a larger study is needed to substantiate this suggestion. Improved parcellation of primary from non-primary cortices could have practical utility in tracking the onset of neurodegenerative pathologies such as Alzheimer’s disease that (within distributed cerebral networks such as the auditory system) tend to target non-primary cortices preferentially (Jacobsen et al., 2015).

### Limitations and Future Directions

Our study has several limitations that suggest directions for further work, with a view, in particular, to enabling extension to clinical dementia populations. This study had no healthy young comparator group: in order to determine the effects of healthy aging *per se* on melody processing and associated brain network function, it will be essential to compare older and younger participant cohorts directly and to stratify the age range of older participant cohorts in a common fMRI paradigm. In order to determine which aspects of the present findings are in fact music-specific, other generic kinds of auditory pattern analysis should be assessed. Within the domain of music, the present study leaves several avenues unexplored, including the role of generic musical ‘rule’ learning over and above the processing of specific melodies and the potential effects of more complex, natural music and different instrumental timbres: these aspects could also be explored in future studies. We did not attempt to control emotional content of the stimulus melodies: this is likely to be a relevant parameter, potentially holding insights into the functional architecture of the healthy older brain as well as neurodegenerative diseases (Hardy et al., 2016). In addition, it will be important to determine the effect of an output task and modulation of attentional set (including the potentially confounding effects of drowsiness) on the activation profiles observed here under passive listening conditions. Finally, if the present findings are to realize their clinical potential, they should be further assessed in a larger cohort of normal older participants and ideally, acquired at different centers and with short-interval re-scanning, in order to assess the reproducibility and robustness of the results: this would provide age-related ‘normative’ data that could then act as a reference for patient populations. The consistent relation between cortical myelin integrity (as indexed by MT value) and activation in primary auditory cortex shown in this study raises the exciting possibility that myeloarchitectural metrics might signal particular functional cortical regions and their viability: however, this requires substantiation through wider application of microstructural MRI techniques, assessment of other cortical areas, correlation with other functional metrics and in particular, extension to clinical populations.

## Conclusion

This study provides new information about the neural substrates of auditory information processing in normal aging, using the paradigm of musical melodies. The findings demonstrate that melody processing is a suitable vehicle for delineating separable neural mechanisms that analyze fundamental attributes (regularity and familiarity) of auditory patterns in the normal older brain. The fMRI signatures identified here corroborate current models of auditory cortical organization derived from younger populations but also suggest certain potential points of divergence that should be addressed in future work. Our findings reaffirm the key role of an auditory association cortical ‘hub’ region (PT) in partitioning different kinds of auditory information, previously delineated in the young normal brain. The findings further suggest that the functional activation of auditory cortex correlates with underlying microarchitectural integrity, measured using a novel structural MRI mapping technique. Moreover, the fMRI signatures of melody processing here sample large-scale, distributed brain networks, previously implicated in the pathogenesis of common dementias. Taken together, these findings underscore the potential utility of musical paradigms for assessing brain network alterations in neurodegenerative disease, referenced to network profiles in the normal older brain. We hope that the present work motivates future clinical applications in this line.

## Author Contributions

JA and HG collected and analysed raw data. Further analysis was performed by MC on MPM data, EB on stimulus properties, and RB on behavioral tests. All authors contributed to designing aspects of this study and writing this original research article.

## Conflict of Interest Statement

The authors declare that the research was conducted in the absence of any commercial or financial relationships that could be construed as a potential conflict of interest.

## Footnotes

1 http://www.fil.ion.ucl.ac.uk/spm

## References

[B1] Agustus J. L. Mahoney C. J. Downey L. E. Omar R. Cohen M. White M. J. (2015). Functional MRI of music emotion processing in frontotemporal dementia. *Ann. N. Y. Acad. Sci.* 1337 232–240. 10.1111/nyas.12620 25773639 PMC4402026

[B2] Ashburner J. (2007). A fast diffeomorphic image registration algorithm. *Neuroimage* 38 95–113. 10.1016/j.neuroimage.2007.07.007 17761438

[B3] Bengtsson S. L. Ullen F. Ehrsson H. H. Hashimoto T. Kito T. Naito E. (2009). Listening to rhythms activates motor and premotor cortices. *Cortex* 45 62–71. 10.1016/j.cortex.2008.07.002 19041965

[B4] Besson M. Schön D. (2001). Comparison between language and music. *Ann. N. Y. Acad. Sci.* 930 232–258. 10.1111/j.1749-6632.2001.tb05736.x 11458832

[B5] Bischoff-Grethe A. Proper S. M. Mao H. Daniels K. A. Berns G. S. (2000). Conscious and unconscious processing of nonverbal predictability in Wernicke’s area. *J. Neurosci.* 20 1975–1981. 10.1523/JNEUROSCI.20-05-01975.2000 10684898 PMC6772930

[B6] Boltz M. G. (1993). The generation of temporal and melodic expectancies during musical listening. *Percept. Psychophys.* 53 585–600. 10.3758/BF03211736 8332426

[B7] Clark C. N. Warren J. D. (2015). Music, memory and mechanisms in Alzheimer’s disease. *Brain* 138 2122–2125. 10.1093/brain/awv148 26205838 PMC4511859

[B8] Cliff M. Joyce D. W. Lamar M. Dannhauser T. Tracy D. K. Shergill S. S. (2013). Aging effects on functional auditory and visual processing using fMRI with variable sensory loading. *Cortex* 49 1304–1313. 10.1016/j.cortex.2012.04.003 22578707

[B9] Costa-Faidella J. Baldeweg T. Grimm S. Escera C. (2011). Interactions between “what” and “when” in the auditory system: temporal predictability enhances repetition suppression. *J. Neurosci.* 31 18590–18597. 10.1523/JNEUROSCI.2599-11.2011 22171057 PMC6623902

[B10] Desikan R. S. Ségonne F. Fischl B. Quinn B. T. Dickerson B. C. Blacker D. (2006). An automated labeling system for subdividing the human cerebral cortex on MRI scans into gyral based regions of interest. *Neuroimage* 31 968–980. 10.1016/j.neuroimage.2006.01.021 16530430

[B11] Dick F. Tierney A. T. Lutti A. Josephs O. Sereno M. I. Weiskopf N. (2012). In vivo functional and myeloarchitectonic mapping of human primary auditory areas. *J. Neurosci.* 32 16095–16105. 10.1523/JNEUROSCI.1712-12.2012 23152594 PMC3531973

[B12] Draganski B. Ashburner J. Hutton C. Kherif F. Frackowiak R. S. Helms G. (2011). Regional specificity of MRI contrast parameter changes in normal ageing revealed by voxel-based quantification (VBQ). *Neuroimage* 55 1423–1434. 10.1016/j.neuroimage.2011.01.052 21277375 PMC3093621

[B13] Fletcher P. D. Warren J. D. (2011). Semantic dementia: a specific network-opathy. *J. Mol. Neurosci.* 45 629–636. 10.1007/s12031-011-9586-3 21710360 PMC3207124

[B14] Friston K. (2005). A theory of cortical responses. *Philos. Trans. R. Soc. Lond. B. Biol. Sci.* 360 815–836. 10.1098/rstb.2005.1622 15937014 PMC1569488

[B15] Gaab N. Schlaug G. (2003). The effect of musicianship on pitch memory in performance matched groups. *Neuroreport* 14 2291–2295. 10.1097/00001756-200312190-00001 14663178

[B16] Goebel R. Khorram-Sefat D. Muckli L. Hacker H. Singer W. (1998). The constructive nature of vision: direct evidence from functional magnetic resonance imaging studies of apparent motion and motion imagery. *Eur. J. Neurosci.* 10 1563–1573. 10.1046/j.1460-9568.1998.00181.x 9751129

[B17] Golden H. L. Agustus J. L. Goll J. C. Downey L. E. Mummery C. J. Schott J. M. (2015). Functional neuroanatomy of auditory scene analysis in Alzheimer’s disease. *Neuroimage Clin.* 7 699–708. 10.1016/j.nicl.2015.02.019 26029629 PMC4446369

[B18] Goll J. C. Crutch S. J. Warren J. D. (2010). Central auditory disorders: toward a neuropsychology of auditory objects. *Curr. Opin. Neurol.* 23 617–627. 10.1097/WCO.0b013e32834027f6 20975559 PMC3374998

[B19] Goll J. C. Kim L. G. Hailstone J. C. Lehmann M. Buckley A. Crutch S. J. (2011). Auditory object cognition in dementia. *Neuropsychologia* 49 2755–2765. 10.1016/j.neuropsychologia.2011.06.004 21689671 PMC3202629

[B20] Goll J. C. Kim L. G. Ridgway G. R. Hailstone J. C. Lehmann M. Buckley A. H. (2012a). Impairments of auditory scene analysis in Alzheimer’s disease. *Brain* 135(Pt 1), 190–200. 10.1093/brain/awr260 22036957 PMC3267978

[B21] Goll J. C. Ridgway G. R. Crutch S. J. Theunissen F. E. Warren J. D. (2012b). Nonverbal sound processing in semantic dementia: a functional MRI study. *Neuroimage* 61 170–180. 10.1016/j.neuroimage.2012.02.045 22405732 PMC3398766

[B22] Grahn J. A. Brett M. (2007). Rhythm and beat perception in motor areas of the brain. *J. Cogn. Neurosci.* 19 893–906. 10.1162/jocn.2007.19.5.893 17488212

[B23] Grahn J. A. Brett M. (2009). Impairment of beat-based rhythm discrimination in Parkinson’s disease. *Cortex* 45 54–61. 10.1016/j.cortex.2008.01.005 19027895

[B24] Grahn J. A. Rowe J. B. (2009). Feeling the beat: premotor and striatal interactions in musicians and nonmusicians during beat perception. *J. Neurosci.* 29 7540–7548. 10.1523/JNEUROSCI.2018-08.2009 19515922 PMC2702750

[B25] Griffiths T. D. Warren J. D. (2002). The planum temporale as a computational hub. *Trends Neurosci.* 25 348–353. 10.1016/S0166-2236(02)02191-4 12079762

[B26] Groussard M. Rauchs G. Landeau B. Viader F. Desgranges B. Eustache F. (2010a). The neural substrates of musical memory revealed by fMRI and two semantic tasks. *Neuroimage* 53 1301–1309. 10.1016/j.neuroimage.2010.07.013 20627131

[B27] Groussard M. Viader F. Hubert V. Landeau B. Abbas A. Desgranges B. (2010b). Musical and verbal semantic memory: two distinct neural networks? *Neuroimage* 49 2764–2773. 10.1016/j.neuroimage.2009.10.039 19854279

[B28] Groussard M. Viader F. Landeau B. Desgranges B. Eustache F. Platel H. (2009). Neural correlates underlying musical semantic memory. *Ann. N. Y. Acad. Sci.* 1169 278–281. 10.1111/j.1749-6632.2009.04784.x 19673793 PMC2909253

[B29] Guéguin M. Le Bouquin-Jeannès R. Faucon G. Chauvel P. Liégeois-Chauvel C. (2007). Evidence of functional connectivity between auditory cortical areas revealed by amplitude modulation sound processing. *Cereb. Cortex* 17 304–313. 10.1093/cercor/bhj148 16514106 PMC2111045

[B30] Hailstone J. C. Omar R. Henley S. M. Frost C. Kenward M. G. Warren J. D. (2009). It’s not what you play, it’s how you play it: timbre affects perception of emotion in music. *Q. J. Exp. Psychol.* 62 2141–2155. 10.1080/17470210902765957 19391047 PMC2683716

[B31] Halpern A. R. Zatorre R. J. (1999). When that tune runs through your head: a PET investigation of auditory imagery for familiar melodies. *Cereb. Cortex* 9 697–704. 10.1093/cercor/9.7.697 10554992

[B32] Hardy C. J. Marshall C. R. Golden H. L. Clark C. N. Mummery C. J. Griffiths T. D. (2016). Hearing and dementia. *J. Neurol.* 263 2339–2354. 10.1007/s00415-016-8208-y 27372450 PMC5065893

[B33] Hardy C. J. D. Agustus J. L. Marshall C. R. Clark C. N. Russell L. L. Bond R. L. (2017a). Behavioural and neuroanatomical correlates of auditory speech analysis in primary progressive aphasias. *Alzheimers Res. Ther.* 9:53. 10.1186/s13195-017-0278-2 28750682 PMC5531024

[B34] Hardy C. J. D. Agustus J. L. Marshall C. R. Clark C. N. Russell L. L. Brotherhood E. V. (2017b). Functional neuroanatomy of speech signal decoding in primary progressive aphasias. *Neurobiol. Aging* 56 190–201. 10.1016/j.neurobiolaging.2017.04.026 28571652 PMC5476347

[B35] Helms G. Dathe H. Kallenberg K. Dechent P. (2008). High-resolution maps of magnetization transfer with inherent correction for RF inhomogeneity and T1 relaxation obtained from 3D FLASH MRI. *Magn. Reson. Med.* 60 1396–1407. 19025906 10.1002/mrm.21732

[B36] Herholz S. C. Coffey E. B. J. Pantey C. Zatorre R. J. (2015). Dissociation of neural networks for predisposition and for training-related plasticity in auditory-motor learning. *Cereb. Cortex* 26 3125–3134. 10.1093/cercor/bhv138 26139842 PMC4898668

[B37] Hsieh S. Hornberger M. Piguet O. Hodges J. R. (2011). Neural basis of music knowledge: evidence from the dementias. *Brain* 134(Pt 9), 2523–2534. 10.1093/brain/awr190 21857031

[B38] Hutton C. Bork A. Josephs O. Deichmann R. Ashburner J. Turner R. (2002). Image distortion correction in fMRI: a quantitative evaluation. *Neuroimage* 16 217–240. 10.1006/nimg.2001.1054 11969330

[B39] Jacobsen J. H. Stelzer J. Fritz T. H. Chételat G. La Joie R. Turner R. (2015). Why musical memory can be preserved in advanced Alzheimer’s disease. *Brain* 138(Pt 8), 2438–2450. 10.1093/brain/awv135 26041611

[B40] Jenkinson M. Beckmann C. F. Behrens T. E. Woolrich W. M. Smith S. M. (2012). FSL. *Neuroimage* 62 782–790. 10.1016/j.neuroimage.2011.09.015 21979382

[B41] Kourtzi Z. Kanwisher N. (2000). Activation in human MT/MST by static images with implied motion. *J. Cogn. Neurosci.* 12 48–55. 10.1162/08989290051137594 10769305

[B42] Kovacevic S. Qualls C. Adair J. C. Hudson D. Woodruff C. C. Knoefel J. (2005). Age-related effects on superior temporal gyrus activity during an auditory oddball task. *Neuroreport* 16 1075–1079. 10.1097/00001756-200507130-00009 15973151

[B43] Leaver A. M. Van Lare J. Zielinski B. Halpern A. R. Rauschecker J. P. (2009). Brain activation during anticipation of sound sequences. *J. Neurosci.* 29 2477–2485. 10.1523/JNEUROSCI.4921-08.2009 19244522 PMC2892726

[B44] Lutti A. Dick F. Sereno M. I. Weiskopf N. (2014). Using high-resolution quantitative mapping of R1 as an index of cortical myelination. *Neuroimage* 93 176–188. 10.1016/j.neuroimage.2013.06.005 23756203

[B45] Lutti A. Hutton C. Finsterbusch J. Helms G. Weiskopf N. (2010). Optimization and validation of methods for mapping of the radiofrequency transmit field at 3T. *Magn. Reson. Med.* 64 229–238. 10.1002/mrm.22421 20572153 PMC3077518

[B46] Lutti A. Thomas D. L. Hutton C. Weiskopf N. (2013). High-resolution functional MRI at 3 T: 3D/2D echo-planar imaging with optimized physiological noise correction. *Magn. Reson. Med.* 69 1657–1664. 10.1002/mrm.24398 22821858 PMC4495253

[B47] Marchant J. L. Driver J. (2013). Visual and audiovisual effects of isochronous timing on visual perception and brain activity. *Cereb. Cortex* 23 1290–1298. 10.1093/cercor/bhs095 22508766 PMC3643713

[B48] Omar R. Hailstone J. C. Warren J. D. (2012). Semantic memory for music in dementia. *Music Percept.* 29 467–477. 10.1525/mp.2012.29.5.467

[B49] Overath T. Cusack R. Kumar S. von Kriegstein K. Warren J. D. Grube M. (2007). An information theoretic characterisation of auditory encoding. *PLoS Biol.* 5:e288. 10.1371/journal.pbio.0050288 17958472 PMC2039771

[B50] Pearce M. (2005). *The Construction and Evaluation of Statistical Models of Melodic Structure in Music Perception and Composition.* Ph.D. thesis, School of Informatics City University, London.

[B51] Pearce M. Wiggins A. (2012). Auditory expectation: the information dynamics of music perception and cognition. *Top. Cogn. Sci.* 4 625–652. 10.1111/j.1756-8765.2012.01214.x 22847872

[B52] Peelle J. E. Troiani V. Wingfield A. Grossman M. (2010). Neural processing during older adults’ comprehension of spoken sentences: age differences in resource allocation and connectivity. *Cereb. Cortex* 20 773–782. 10.1093/cercor/bhp142 19666829 PMC2837088

[B53] Pereira C. S. Teixeira J. Figueiredo P. Xavier J. Castro S. L. Brattico E. (2011). Music and emotions in the brain: familiarity matters. *PLoS One* 6:e27241. 10.1371/journal.pone.0027241 22110619 PMC3217963

[B54] Peretz I. Coltheart M. (2003). Modularity of music processing. *Nat. Neurosci.* 6 688–691. 10.1038/nn1083 12830160

[B55] Peretz I. Gosselin N. Belin P. Zatorre R. J. Plailly J. Tillmann B. (2009). Music lexical networks: the cortical organization of music recognition. *Ann. N. Y. Acad. Sci.* 1169 256–265. 10.1111/j.1749-6632.2009.04557.x 19673789

[B56] Peretz I. Kolinsky R. Tramo M. Labrecque R. Hublet C. Demeurisse G. (1994). Functional dissociations following bilateral lesions of auditory cortex. *Brain* 117(Pt 6), 1283–1301. 10.1093/brain/117.6.1283 7820566

[B57] Pievani M. de Haan W. Wu T. Seeley W. W. Frisoni G. B. (2011). Functional network disruption in the degenerative dementias. *Lancet Neurol.* 10 829–843. 10.1016/S1474-4422(11)70158-2 21778116 PMC3219874

[B58] Plailly J. Tillmann B. Royet J.-P. (2007). The feeling of familiarity of music and odors: the same neural signature? *Cereb. Cortex* 17 2650–2658. 10.1093/cercor/bhl173 17289777

[B59] Platel H. Baron J. C. Desgranges B. Bernard F. Eustache F. (2003). Semantic and episodic memory of music are subserved by distinct neural networks. *Neuroimage* 20 244–256. 10.1016/S1053-8119(03)00287-8 14527585

[B60] Platel H. Price C. Baron J. C. Wise R. Lambert J. Frackowiak R. S. (1997). The structural components of music perception. A functional anatomical study. *Brain* 120(Pt 2), 229–243. 10.1093/brain/120.2.229 9117371

[B61] Profant O. Tintěra J. Balogová Z. Ibrahim I. Jilek M. Syka J. (2015). Functional changes in the human auditory cortex in ageing. *PLoS One* 10:e0116692. 10.1371/journal.pone.0116692 25734519 PMC4348517

[B62] Rimmele J. Schröger E. Bendixen A. (2012). Age-related changes in the use of regular patterns for auditory scene analysis. *Hear. Res.* 289 98–107. 10.1016/j.heares.2012.04.006 22543088

[B63] Rohrer J. D. Lashley T. Schott J. M. Warren J. E. Mead S. Isaacs A. M. (2011). Clinical and neuroanatomical signatures of tissue pathology in frontotemporal lobar degeneration. *Brain* 134(Pt 9), 2565–2581. 10.1093/brain/awr198 21908872 PMC3170537

[B64] Saito Y. Ishii K. Sakuma N. Kawasaki K. Oda K. Mizusawa H. (2012). Neural substrates for semantic memory of familiar songs: is there an interface between lyrics and melodies? *PLoS One.* 7:e46354. 10.1371/journal.pone.0046354 23029492 PMC3460812

[B65] Satoh M. Takeda K. Nagata K. Hatazawa J. Kuzuhara S. (2003). The anterior portion of the bilateral temporal lobes participates in music perception: a positron emission tomography study. *AJNR Am. J. Neuroradiol.* 24 1843–1848. 14561614 PMC7976296

[B66] Satoh M. Takeda K. Nagata K. Shimosegawa E. Kuzuhara S. (2006). Positron-emission tomography of brain regions activated by recognition of familiar music. *AJNR Am. J. Neuroradiol.* 27 1101–1106. 16687552 PMC7975750

[B67] Sereno M. I. Lutti A. Weiskopf N. Dick F. (2013). Mapping the human cortical surface by combining quantitative T(1) with retinotopy. *Cereb. Cortex* 23 2261–2268. 10.1093/cercor/bhs213 22826609 PMC3729202

[B68] Sikka R. Cuddy L. L. Johnsrude I. S. Vanstone A. D. (2015). An fMRI comparison of neural activity associated with recognition of familiar melodies in younger and older adults. *Font. Neurosci.* 9:356. 10.3389/fnins.2015.00356 26500480 PMC4594019

[B69] Snyder J. S. Alain C. (2007). Sequential auditory scene analysis is preserved in normal aging adults. *Cereb. Cortex* 17 501–512. 10.1093/cercor/bhj175 16581981

[B70] Teki S. Grube M. Griffiths T. D. (2011a). A unified model of time perception accounts for duration-based and beat-based timing mechanisms. *Front. Integr. Neurosci.* 5:90. 10.3389/fnint.2011.00090 22319477 PMC3249611

[B71] Teki S. Grube M. Kumar S. Griffiths T. D. (2011b). Distinct neural substrates of duration-based and beat-based auditory timing. *J. Neurosci.* 31 3805–3812. 10.1523/JNEUROSCI.5561-10.2011 21389235 PMC3074096

[B72] Warren J. D. Bamiou D. E. (2018). Prevention of dementia by targeting risk factors. *Lancet* 391:1575 10.1016/S0140-6736(18)30579-8 29695344

[B73] Warren J. D. Fletcher P. D. Golden H. L. (2012). The paradox of syndromic diversity in Alzheimer disease. *Nat. Rev. Neurol.* 8 451–464. 10.1038/nrneurol.2012.135 22801974

[B74] Warren J. D. Griffiths T. D. (2003). Distinct mechanisms for processing spatial sequences and pitch sequences in the human auditory brain. *J. Neurosci.* 23 5799–5804. 10.1523/JNEUROSCI.23-13-05799.2003 12843284 PMC6741275

[B75] Warren J. D. Rohrer J. D. Rossor M. N. (2013a). Clinical review. Frontotemporal dementia. *BMJ* 347:f4827. 10.1136/bmj.f4827 23920254 PMC3735339

[B76] Warren J. D. Rohrer J. D. Schott J. M. Fox N. C. Hardy J. Rossor M. N. (2013b). Molecular nexopathies: a new paradigm of neurodegenerative disease. *Trends Neurosci.* 36 561–569. 10.1016/j.tins.2013.06.007 23876425 PMC3794159

[B77] Warren J. E. Wise R. J. Warren J. D. (2005). Sounds do-able: auditory-motor transformations and the posterior temporal plane. *Trends Neurosci.* 28 636–643. 10.1016/j.tins.2005.09.010 16216346

[B78] Watson J. D. Myers R. Frackowiak R. S. Hajnal J. V. Woods R. P. Mazziotta J. C. (1993). Area V5 of the human brain: evidence from a combined study using positron emission tomography and magnetic resonance imaging. *Cereb. Cortex* 3 79–94. 10.1093/cercor/3.2.79 8490322

[B79] Weiskopf N. Hutton C. Josephs O. Deichmann R. (2006). Optimal EPI parameters for reduction of susceptibility-induced BOLD sensitivity losses: a whole-brain analysis at 3 T and 1.5 T. *Neuroimage* 33 493–504. 10.1016/j.neuroimage.2006.07.029 16959495

[B80] Weiskopf N. Lutti A. Helms G. Novak M. Ashburner J. Hutton C. (2011). Unified segmentation based correction of R1 brain maps for RF transmit field inhomogeneities (UNICORT). *Neuroimage* 54 2116–2124. 10.1016/j.neuroimage.2010.10.023 20965260 PMC3018573

[B81] Weiskopf N. Suckling J. Williams G. Correia M. M. Inkster B. Tait R. (2013). Quantitative multi-parameter mapping of R1, PD(^∗^), MT, and R2(^∗^) at 3T: a multi-center validation. *Front. Neurosci.* 7:95 10.3389/fnins.2013.00095 PMC3677134 23772204

[B82] Wise R. J. Scott S. K. Blank S. C. Mummery C. J. Murphy K. Warburton E. A. (2001). Separate neural subsystems within ‘Wernicke’s area’. *Brain* 124(Pt 1), 83–95. 10.1093/brain/124.1.83 11133789

[B83] Wong P. C. Jin J. X. Gunasekera G. M. Abel R. Lee E. R. Dhar S. (2009). Aging and cortical mechanisms of speech perception in noise. *Neuropsychologia* 47 693–703. 10.1016/j.neuropsychologia.2008.11.032 19124032 PMC2649004

[B84] Zhou J. Greicius M. D. Gennatas E. D. Growdon M. E. Jang J. Y. Rabinovici G. D. (2010). Divergent network connectivity changes in behavioural variant frontotemporal dementia and Alzheimer’s disease. *Brain* 133 1352–1367. 10.1093/brain/awq075 20410145 PMC2912696

