# Supplementary material for: Melody Processing Characterizes Functional Neuroanatomy in the Aging Brain
Source: Front Neurosci. 2018 Nov 19;12:815. doi: 10.3389/fnins.2018.00815 (PMC6262413; doi:10.3389/fnins.2018.00815)
Supplement: Supplementary file 1 [file Table_1.PDF]

Supplementary Table S1

The twenty-four melodies used in the familiar anisochronous (**FA**) condition, with relevant stimulus

**Composer**	**Musical piece**	**No. of notes**	**Pitch range**	**Pitch change std (semitones)***
Bizet	Toreador's Song from Carmen	18	C1 - D2	3.18
Bizet	Habanera from Carmen (excerpt 1)	35	D1 - C2#	2.40
Bizet	Habanera from Carmen (excerpt 2)	29	D1 - D2	2.23
Boccherini	Minuet from String Quintet in E	30	D1 - A2	4.12
Brahms	Hungarian Dance No 5	19	F1# - A2	3.27
Charpentier	Prelude from Te Deum	21	G1 - G2	2.87
Delibes	Mazurka from Coppelia	21	G1 - C3	4.61
Dvorak	Humoreske (excerpt 1)	21	G1 - B2	3.35
Dvorak	Humoreske (excerpt 2)	28	C1 - A2	2.70
Handel	Hornpipe from the Water Music	28	C1 - G1	2.87
Joplin	The Entertainer (excerpt 1)	36	D1 - E2	5.14
Joplin	The Entertainer (excerpt 2)	48	D1 - E2	2.60
Mozart	Eine Kleine Nachtmusik	18	D1 - D2	4.25
Mozart	Piano Concerto No 21, 2nd Mov	26	C2 - D3	4.68
Mozart	Symphony No 40, 1st Mov (excerpt 1)	40	C1 - A1#	2.86
Mozart	Symphony No 40, 1st Mov (excerpt 2)	31	C2# - C3	2.96
Mozart	Turkish Rondo from Piano Sonata No 11	43	G1# - C3	2.35
Prokofiev	Peter and the Wolf	25	G1 - C3	4.28
Ravel	Bolero	33	C1 - D2	1.99
Strauss	Radetsky March	43	C1 - B1	3.34
Strauss	Tritsch Tratsch Polka	38	B0 - E2	5.10
Tchaikovsky	Waltz of the Flowers from The Nutcracker	27	F1 - F2	4.17
Vivaldi	Spring from The Four Seasons	32	B1 - B2	3.03
Wagner	Ride of the Valkyries	18	F0# - A1	5.02
Mean:		*29.5*		*3.47*

*standard deviation for inter-tone pitch variation across the 8 second excerpt
