# Supplementary material for: Melody Processing Characterizes Functional Neuroanatomy in the Aging Brain
Source: Front Neurosci. 2018 Nov 19;12:815. doi: 10.3389/fnins.2018.00815 (PMC6262413; doi:10.3389/fnins.2018.00815)
Supplement: Supplementary file 2 [file Table_2.pdf]

Supplementary Table S2

The twenty-four melodies used in the familiar isochronous (**FI**) condition, with relevant stimulus parameters.

**Composer**	**Musical Piece**	**Total notes**	**Pitch range**	**Pitch change std (semitones)***
Bach	Fugue in D minor	32	A0 - A1	7.07
Bach	Jesu Joy of Man's Desiring	32	D1 - G2	2.87
Bach	Minuet in G (excerpt 1)	32	A1 - E2	1.67
Bach	Minuet in G (excerpt 2)	32	F1# - G2	3.91
Bach	Toccata in D minor	32	F1 - A2#	2.58
Barber	Adagio for Strings	16	A1 - D2#	1.84
Beethoven	Fur Elise (excerpt 1)	32	E0 - E3	8.44
Beethoven	Fur Elise (excerpt 2)	32	E0 - E2	6.74
Beethoven	Moonlight Sonata	32	G0# -F1#	5.93
Beethoven	Ode to Joy	32	D1 - E2	3.67
Delibes	Flower Duet from Lakme	32	G1 - D2#	1.74
Dvorak	New World Symphony, Adagio	16	F1 - C2	2.42
Grieg	Morning Mood from Peer Gynt (excerpt 1)	32	F1 - D2	2.95
Grieg	Morning Mood from Peer Gynt (excerpt 2)	32	E1 - C2#	3.43
Grieg	Mountain King from Peer Gynt (excerpt 1)	32	C1# - A1	3.03
Grieg	Mountain King from Peer Gynt (excerpt 2)	32	B0 - B1	3.60
Handel	Arrival of the Queen of Sheba (excerpt 1)	32	B0 - E2	2.35
Handel	Arrival of the Queen of Sheba (excerpt 2)	32	G1 - G2	3.79
Offenbach	Infernal Gallop from Orpheus (excerpt 1)	32	A1 - D3	3.94
Offenbach	Infernal Gallop from Orpheus (excerpt 2)	32	G1 - G2	3.20
Puccini	Nessum Dorma	16	B1 - G2	2.52
Quilter	Upon St Paul's	16	D1 - D2	4.72
Saint-Saens	Danse Macabre	32	D1 - A1#	2.26
Tchaikovsky	Dance of the Little Swans from Swan Lake	32	F0# -F2#	4.84
Mean:		*29.3*		*3.73*

*standard deviation for inter-tone pitch variation across the 8 second excerpt

Melodies in the FA and FI conditions did not differ significantly in mean tempo or pitch variation ($p > 0.1$).
