# Supplementary material for: Melody Processing Characterizes Functional Neuroanatomy in the Aging Brain
Source: Front Neurosci. 2018 Nov 19;12:815. doi: 10.3389/fnins.2018.00815 (PMC6262413; doi:10.3389/fnins.2018.00815)
Supplement: Supplementary file 3 [file Table_3.PDF]

Supplementary Methods & Results

Peripheral hearing was assessed in 14 out of 20 participants (7 females) using a pure-tone audiometry procedure administered to the right ear via headphones from a notebook computer (Golden et al., 2015). Participants were instructed to indicate as soon as they could detect a continuous tone that was linearly increasing in intensity from 0dB to 80dB across a period of 1 minute 20 seconds. Four frequencies (500, 1000, 2000 and 4000 Hz) were tested and each repeated three times. An overall mean detection threshold (dB) averaged across the four frequencies was compared to age-matched normed data and a z-score generated for each participant. All participants produced negative z-scores, indicating that they all performed better (i.e. threshold intensity was lower) than the mean population without hearing aids (see table below). The group mean z-score was -1.05 ranging from -1.91 to -0.91.

Sex	Age at testing	Overall intensity threshold (dB)	Z-score using age-matched normed data
F	61	10.67	-0.69
F	62	16.51	-0.19
F	65	7.93	-0.94
F	68	5.67	-1.15
F	69	2.73	-1.42
F	73	12.26	-1.39
F	78	17.68	-1.08
M	56	13.05	-0.50
M	65	2.02	-1.91
M	67	16.44	-0.91
M	67	9.35	-1.40
M	69	20.23	-0.65
M	70	6.80	-1.58
M	73	24.29	-0.83
